# A Video-Based Tactical Task Does Not Elicit Mental Fatigue and Does Not Impair Soccer Performance in a Subsequent Small-Sided Game

**DOI:** 10.3390/sports10030031

**Published:** 2022-02-27

**Authors:** Gianmarco Ciocca, Antonio Tessitore, Mauro Mandorino, Harald Tschan

**Affiliations:** 1 Centre for Sports Science and University Sports, University of Vienna, Auf der Schmelz 6 (USZ I), 1150 Vienna, Austria; harald.tschan@univie.ac.at; 2 Department of Movement, Human and Health Sciences, University of Rome “Foro Italico”, Piazza L. de Bosis 6, 00135 Rome, Italy; antonio.tessitore@uniroma4.it (A.T.); m.mandorino@studenti.uniroma4.it (M.M.); 3 Performance and Analytics Department, Parma Calcio 1913, 43121 Parma, Italy

**Keywords:** mental effort, decision making, cognitive, perception of effort, motivation, football

## Abstract

Mental fatigue can impair physical, technical, and tactical performance in sports. Since most previous research used general cognitive tasks to elicit mental fatigue, the aim of this study was to investigate whether a more sport-specific task could induce the effects of mental fatigue and impair the subsequent physical and technical performance in a soccer small-sided game. Ten soccer players performed two small-sided games on two different days in a crossover design. Before each small-sided game, they performed a video-based tactical task (30 min) and a control task (documentary watching, 30 min) in a randomized and counterbalanced order. Mental effort was measured through a visual analog scale after the tactical and control tasks. Subjective ratings of perceived exertion were assessed through the RPE questionnaire after the end of the SSG. Physical performance was assessed during the SSG through GPS technology. Results showed no differences (*p* > 0.05) in physical performance between the two conditions. None of the technical variables were negatively affected by the video-based tactical condition, with the number of total passes (*p* = 0.003; ES = 0.72 medium) and successful passes (*p* = 0.003; ES = 0.82 large) results even improved by the video-based tactical task. The mental effort required by the video-tactical task was significantly higher than the control task (*p* = 0.002; ES = 2.09 huge). However, overall RPE did not differ between conditions. The video-based tactical task did not elicit mental fatigue and did not impair subsequent physical and technical performance. The higher ecological validity of the task and the higher motivation of the participants might have contributed to the results.

## 1. Introduction

Situational team sports such as soccer are complex activities involving a combination of physical, cognitive, tactical, psychological, and emotional skills [1,2,3,4]. Such activities require players to constantly monitor the dynamic and ever-changing game environment, retrieve and analyze the most relevant information to quickly make the most appropriate choice, according to the teammates and opponents’ behaviors and other space–time variables.

Considering the multifactorial genesis of fatigue beyond the only metabolic and neuromuscular perspective [5], several authors highlighted the cognitive demands of play, as well the magnitude of their loads in relation to the onset of fatigue [6,7]. In fact, managing such complex situations during an intense and prolonged physical activity classifies soccer and other situational team sports as mentally fatiguing activities [6,8,9], to the point that such performances have also been defined as the “brain’s biggest challenge” [8,10]. 

Mental fatigue has been defined as a psychobiological state caused by prolonged periods of demanding cognitive activity [11], which may result in an acute increase in subjective ratings of fatigue and/or an acute decline in cognitive and/or physical performance [5,12,13]. Pattyn et al. [14] reported the evolution of fatigue’s proposed models over the years where the perception of effort is recognized as playing a central role, possibly due to neurophysiological alterations such as an unbalanced presence of different neurotransmitters in specific brain areas, caused by prolonged mental exertion [6,12,13,14]. 

In the literature, the investigation of the impact of cognitive fatigue on subsequent physical performance has yielded controversial results. Schucker and MacMahon [15] showed how both a cognitively fatiguing task (unmatched Stroop for 10 min) and an easy cognitive task (matched Stroop for 10 min or watching two 5 min videos containing nature scenes) failed to impair the performance of a standardized shuttle run. Previously, through a longer (90 min) computer fatiguing task, Marcora et al. [11] showed that mentally fatigued subjects reached their maximal level of perceived exertion and disengaged from the physical task earlier than the control group. The relationship between cognitive aspects and performance is then mediated by different important factors, such as participants’ level of experience with the task and their physical training, specific to the performance task.

In soccer, as an effect of induced mental fatigue by using a 30 min Stroop test, an impairment of soccer-specific physical (reduced running distance) and technical (passing and shooting) performances were reported [16], as well as impaired decision-making ability [17]. In the latter study, players were required to watch film-based simulations of offensive soccer play on a screen and then make quick and accurate decisions by performing the appropriate action (i.e., passing to any player on screen, shooting toward goal, or dribbling the ball around a defender) [17]. Results of that study indicated that mental fatigue worsened the speed and accuracy of soccer-specific decision making, possibly due to an impaired player’s capability to use environmental cues and/or changes in attention and decision-making strategies. Regarding the duration of the cognitive fatiguing task, Gantois et al. [18] demonstrated that a shorter (15 min) cognitive Stroop task was insufficient in contrast to a longer one (30 min) to affect passing decision making during a 90 min soccer match simulation. In line with these findings, the use of a computer-based [19], paper colored version [20], and the smartphone app version [21] of a 30 min Stroop task protocol showed as mental fatigue affected physical activity (i.e., distance in acceleration covered per minute), as well as offensive and defensive technical performance (i.e., passing accuracy, interceptions, tackles, shots, dribbles), decision making, and perception of effort (RPE) in subsequent soccer small-sided games (SSGs). Moreover, in a study including peripheral perception factors, Kunrath et al. [7] reported how mental fatigue elicited by 30 min of a modified Stroop test affected players’ peripheral perception ability and tactical behavior on a subsequent soccer SSG. This study also showed that the players covered greater distances at low velocities to counterbalance their poor tactical decisions [7]. Using a different approach instead of adopting a laboratory condition such as the Stroop test, Fortes et al. [22] showed that also common daily practices such as using smartphones for social networks and playing videogames elicited mental fatigue and impaired passing decision making in soccer players. 

However, in all studies cited above, mentally fatiguing tasks were based on a general and not a sport-specific task. In fact, several authors underlined that further steps toward a sport-specific and ecological valid test design would be required [6,9,13]. 

Since decision-making and tactical abilities play relevant roles in situational team sports, they are also trained and stimulated besides field activities. Specifically, video-based tactical tasks are used as a perceptual–cognitive assessment tool and training method for improving decision making and tactical knowledge [23,24,25,26,27,28]. During these tasks, players are required to provide tactical solutions (verbally, writing, or performing a short movement) to the presented game situations on a screen, involving decision making, sport-specific working memory, and sustained attention [28]. Moreover, they are commonly used by coaching staff to inform the entire team or individual players about the playing characteristics of opponents, during the training week or before the match itself.

Therefore, the aim of this study was to investigate whether a more soccer-specific task requiring more sport-specific attention and decision-making skills (compared with the general psychological Stroop test) in the form of a video-based tactical test could induce a level of mental fatigue that would impair the subsequent physical and technical performance in a soccer SSG. Based on previous research, we hypothesized that the video-based task would result in mental fatigue and a subsequent decrease in soccer-specific performance.

## 2. Materials and Methods

### 2.1. Participants

Ten (10) soccer players (age: 17.2 years ± 0.9; height: 178.2 ± 6.5 cm; body mass: 74.4 ± 8.2 kg) competing at the national level in an Under-18 Italian league participated in this study. Participants had no previous muscular injures in the last 60 days before the testing sessions. All subjects and the legal tutors of minor participants were informed about the possible benefits and risks related to their participation and signed an informed consent form before proceeding to any physical test. The study was designed in fulfillment of the ethical guidelines communicated in the Declaration of Helsinki and approved by the host institution’s local ethical committee (Reference Number: 00598). 

### 2.2. Study Design and Experimental Conditions

According to their playing positions, participants were randomly divided into two groups and performed the two experimental conditions (video-based tactical (VT) task; control, (C) task) in a randomized, counterbalanced, crossover design. One group (5 players) performed the VT task on the first session day and the C task on the second session day, while the other group (5 players) performed the opposite. Both conditions lasted 30 min, which is the same duration as the non-sport-specific mentally fatiguing tasks (Stroop test) used in previous studies [16,19,21]. 

**Figure 1:**
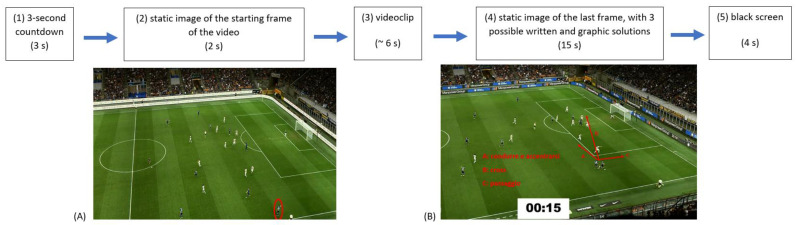
Schematic representation of the sequence for each clip. An exemplifying representation is provided for the static starting frame of a clip (**A**), and the static final frame of the clip displaying the possible solutions (**B**).

In the VT condition, participants were required to watch a video clip with a game situation on a screen and provide the best tactical solution for the game continuation (e.g., *“short pass to the defender”, “move forward”, “shoot”, “long pass to the striker”,* etc.) once the video stopped (temporal occlusion). The test was composed of 60 video clips, of which 30 displayed defensive situations and 30, offensive situations. The videoclips were extrapolated from Italian first league matches (“Serie A”) recorded with cameras placed on the top of stadiums (called “tactic camera”), allowing a top view of the soccer pitch and all the players. The choice of game situations to be extrapolated was made in accordance with the tactical principle of soccer [29,30,31]. Each clip was structured in the following order [31]: (1) a 3 s countdown appeared on the screen; (2) a static image of the starting frame of the video was shown for 2 s, with the player object of the subsequent question circled in red; (3) a video clip of ~6 s was shown; (4) the video stopped to display a static image of the last frame for 15 s with three possible written and graphic solutions (e.g., A: pass the ball to the right; B: shoot; C: pass the ball to the left); (5) the screen became black before starting the next countdown (Figure 1). Therefore, participants had 15 s to make the right choice and manually tick the answer with a pen on paper (A, B, or C). Correct answers or personal scores were not provided to the players at the end of the session.

The C condition consisted of watching a documentary specifically chosen to have the same duration as the VT condition. 

### 2.3. Procedures, Experimental Protocol, and Measurements

The two testing days were performed at the same time in the afternoon, on the same artificial-grass soccer field where the team used to train and compete and interspersed by 24 h, during which players observed a rest day. Before the experimental days, participants underwent two specific familiarization sessions with all procedures and tasks. 

On the day of the experiment, after the arrival at the training facility, all participants were assessed for their motivation (PRE-TASK) using a 100 mm visual analog scale (VAS). Then, participants were randomly divided into two groups, one performing the VT and the other performing the C condition. After that, all participants completed another 100 mm VAS to assess the mental effort (POST-TASK). Participants were again assessed for their motivation for the upcoming SSG (PRE-SSG) using a 100 mm VAS. Then, after a 10 min long physical and technical warm-up, participants performed a 5 vs. 5 SSG (field size 26 × 36 m), divided into 2 halves of 7 min each with 1 min half-time, as prescribed in Trecroci et al. [21]. Differently from Trecroci et al. [21], there were no goalkeepers in the present study, with the players that had to score in two small goals (1 m × 1 m). At the end of the SSG, players were assessed for their overall perceived fatigue and exertion using the Borg’s CR10 Rating of Perceived Exertion (RPE) scale, according to previous research [16,22], and because the players already had a strong familiarity with the questionnaire that was routinely used for their training activities.

The physical data during the SSG were collected using Johan GPS technology (JOHAN Sports, Noordwijk, The Netherlands), consisting of a GPS sensor (10 Hz, including EGNOS correction), accelerometer, gyroscope, and magnetometer (100 Hz, 3 axes, ±16 g). Each device was placed between the players’ scapulae through a tight vest. All GPS devices were turned on before the 10 min warm-up to ensure an optimal signal acquisition. To avoid interunit variability, each player wore the same GPS device during both testing days. Motion data from the trackers were uploaded after each session to the JOHAN Sports online analysis platform. The JOHAN software was used to process the data. The processing was executed using 1 s data resolution (aggregated from 10 Hz motion data). The data captured by trackers during the 1 min break between SSG’s halves were cut and excluded.

The following physical activity measures were collected: total distance, distance covered at walking speed (0–7 km/h), distance covered at low speed (7–14 km/h), distance covered at high speed (14–20 km/h), distance covered at very high speed (>20 km/h), number of accelerations (>2 m/s^2^), number of decelerations (>−2 m/s^2^), and player load. Player load is a measure of the instantaneous rate of change in accelerations in the anteroposterior, mediolateral, and craniocaudal axes, that quantifies the physical loads placed on the athletes [32,33]. 

The SSGs were recorded using a video camera (GoPro Hero Black 5, GoPro, San Mateo, CA, USA), and the footage was used to assess the players’ technical performance, through a notational analysis of the following parameters: 

(1) Passes: total number of passes, successful passes (passes reaching the intended teammate unobstructed), negative passes (passes not reaching the teammate), and passing accuracy (defined as the percentage of successful passes with respect to the total number);

(2) Tackles: total number of tackles, successful tackles (the opposing player was dispossessed of the ball), negative tackles (the opposing player maintained the possession of the ball), and tackling success (defined as the percentage of successful tackles with respect to the total number);

(3) Shots: total number of shots, successful shots (shot resulting in the goal), negative shots (shots not resulting in the goal), and shooting accuracy (defined as the percentage of shots that resulted in a goal with respect to the total number); 

(4) The number of control errors (defined as any non-passing error resulting in loss of possession).

The VAS used to assess motivation and mental effort were anchored at one end with “none at all” and at the other end with “maximal”. Participants were instructed to mark a vertical line anywhere along the 100 mm scale to reflect their current state.

**Figure 2:**
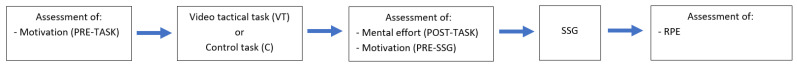
Schematic representation of all procedures for both testing days.

Figure 2 summarizes the order of all procedures for each group and on each testing day.

### 2.4. Statistical Analysis

Statistical analyses were performed using SPSS software (Version 26.0, SPSS Inc., Chicago, IL, USA). Data are presented as mean ± SD. Cohen’s d was used to calculate effect sizes (ES), and results were interpreted as follows: 0.01–0.2 very small, 0.21–0.5 small, 0.51–0.8 medium, 0.81–1.2 large, 1.21–2.0 very large, >2.0 huge [34]. The level of significance was set at *p* ≤ 0.05, with all calculations based on a 95% confidence interval (CI). Shapiro–Wilk test was applied to assess the normality of data. Paired sample *t*-tests were applied to assess differences for mental effort (POST-TASK), RPE, and all normally distributed technical and physical variables between VT and C conditions. Nonparametric Wilcoxon signed ranks tests were applied for non-normally distributed variables (successful shots, negative shots, shooting accuracy, and control errors).

A two-way (2 × 2) repeated measures ANOVA was applied (condition (VT, C) × time (PRE-TASK, PRE-SSG)) to analyze differences in motivation between the two conditions and the two time points (before and after the VT or C tasks). Mauchly’s test was carried out and Greenhouse–Geisser correction was applied if sphericity was violated. 

## 3. Results

**Figure 3:**
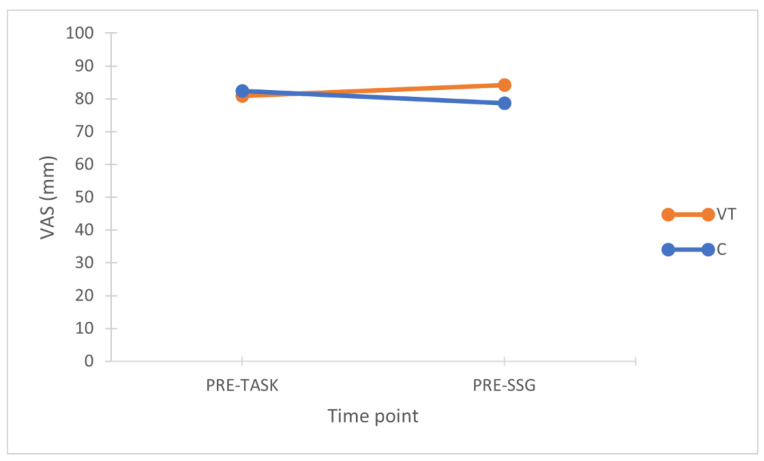
Mean motivation values before (PRE-TASK) and after VT and C tasks (before the SSG). VT = video-based tactical condition; C = control condition.

**Figure 4:**
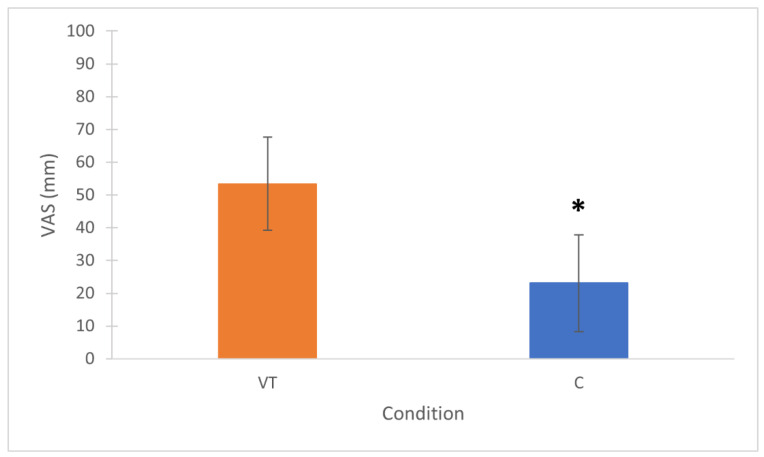
Mean values ± SD of perceived mental effort after VT and C tasks. VT = video-based tactical condition; C = control condition. * *p* = 0.002; ES = 2.09 huge.

**Figure 5:**
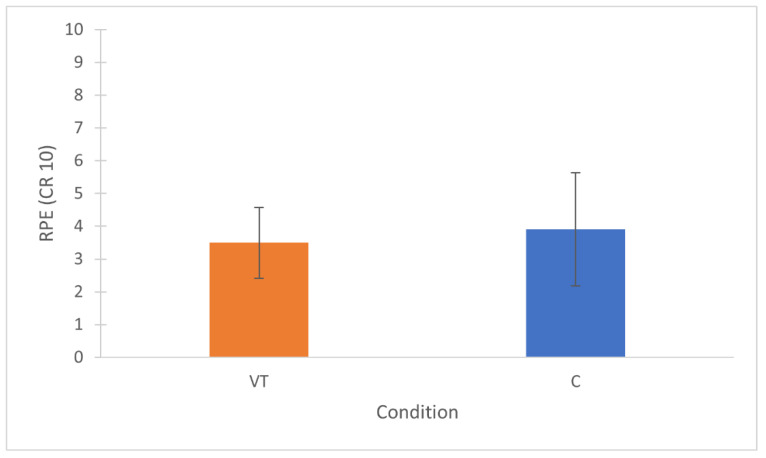
Mean times ± SD of RPE values collected after the SSG. VT = video-based tactical condition; C = control condition.

The two-way (2 × 2) repeated measures ANOVA reported no differences in motivation for the interaction condition × time (*p* = 0.275; η_p_^2^ = 0.131) (Figure 3). The mental effort (POST-TASK) required by the two tasks was significantly higher in the VT condition (*p* = 0.002; ES = 2.09 huge) (Figure 4). All physical activity variables did not differ between conditions (Table 1). Total passes (*p* = 0.003; ES = 0.72 medium) and successful passes (*p* = 0.003; ES = 0.82 large) were significantly higher in VT, while all other technical variables did not differ between conditions (Table 2). RPE values collected at the end of the SSG did not differ between conditions (VT = 3.5 ± 0.8; C = 3.9 ± 1.7; *p* = 0.591; ES = 0.23 small) (Figure 5). 

## 4. Discussion

The purpose of this investigation was to examine the effects of induced mental fatigue on the performance of soccer SSG. It was hypothesized that a pre-exposition to a soccer-specific fatiguing task would impair players’ physical and technical performance in a subsequent SSG. 

Contrary to our hypothesis, the main finding of this study was that a 30 min video-based tactical task did not elicit mental fatigue and did not impair any of the physical and technical variables in a subsequent soccer SSG, which is not in line with previous studies that used the general Stroop test [7,19,20,21]. Although the mental effort required by the tactical task was rated consistently higher than that required by the control task (*p* = 0.002; ES = 2.09 large) (Figure 4), the RPE collected after the SSG revealed no differences between the two conditions, meaning that players eventually perceived both overall conditions (PRE-TASK + SSG) as equally fatiguing (Figure 5). This aspect also supports the findings of previous research, which showed that an increased perception of effort is the primary indicator and medium of performance impairments related to mental fatigue [12,13,14,19,21]. 

Regarding the physical activity profiles, our results revealed no significant differences between the two conditions. This is in contrast with the studies of Trecroci et al. [21] and Kunrath et al. [7] but in line with the study of Badin et al. [19]. 

Regarding the technical performance, the video-based tactical test submitted in our study not only failed to elicit mental fatigue and impair the performance as that recorded in previous investigations using the Stroop test (Ref. [19] for passing accuracy, control errors, and tackling success; Ref. [21] for passing and shooting accuracy; Ref. [20] for passing and shooting accuracy, and control errors), but it even improved the number of total passes (*p* = 0.003; ES = 0.72 medium) and the number of successful passes (*p* = 0.003; ES = 0.82 large) (Table 2). Although it did not reach statistical significance (*p* = 0.055), the improvement of the passing accuracy was noteworthy (ES = 0.95 large). 

There are several reasons that could explain the absence of impairments of the performance in our study, compared with previous ones using the Stroop test. Firstly, even though video-based tasks involve sport-specific working memory, attention, and decision making [28], the solutions to be provided required a deeper and more complex context analysis due to the multiple elements in the clip, while the psychological Stroop test involves more inhibitory functions [6,35]. Indeed, boring tasks with low information load that require response inhibitions have been shown to induce mental fatigue for longer periods of time [35]. Moreover, although the demands of our tactical test involving 12 tactical principles of soccer [29,31] included a higher variety of tasks, it is possible that the higher number of repetitions/trials that have to be performed in the Stroop makes it more mental fatiguing. In fact, during the 30 min of our video-based tactical test, the players were required to provide 60 solutions (one every 30 s), while during 30 min of Stroop, participants are required to give ~720 solutions (one every ~2.5 s) [35]. In addition, the tasks of a sport-specific cognitive tactical test are certainly more familiar and well-known to the players than those of the Stroop test, which is not an ordinary activity, and it is not performed outside laboratory environments or for training activities [23,24,28]. Finally, and importantly, high levels of motivation have been proven to help avoid performance impairments under fatigue conditions [13,14,35,36,37]. This might be the case in our study, where participants reported levels of motivation (Figure 3) that were considerably higher than those recorded in previous studies using the Stroop test before a soccer SSG [19,21]. In line with this statement, Soylu et al. [20] reported that the Stroop test negatively affects the enjoyment of subsequent physical activities. Therefore, the high level of motivation perceived by participants in our study might have contributed to the maintenance of performance and the avoidance of impairments, as well as to the improvements detected in passing technical performance (Table 2). 

In fact, considering that video-based tactical training sessions have been shown to be effective in improving specific sports performances [23,25,26,28], and also according to the findings of a review article of McGowan et al. [38] regarding the positive effects of psychological mechanisms that can be driven by some warm-up strategies, it can be speculated that our participants acutely benefited from the tactical tasks of the video exposition during the pre-SSG phase as a form of mental preparation for the upcoming SSG. Watching or fulfilling tactical video-based tasks immediately before exercising as video-primed mental stimulation is frequently used in some sports (mainly individual sports). Mental priming might have prepared players for action and optimized their readiness in subsequent SSG. In fact, mental priming is defined as a process in which exposure to a stimulus activates relevant mental representations that are given increased weight in subsequent judgment tasks [39]. In the current study, this might have helped players in optimizing their pre-exercise mindset, which finally resulted in improved playing performance. The presence of this mental-priming effect raised by the video-based tactical task may also be supported by the findings of Fortes et al. [22], in which even a passive, general, and neutral exposure to smartphones and social networks elicited mental fatigue and impaired passing decision making in subsequent soccer SSGs. Moreover, participants exposed to tasks delivered on smartphones might also have experienced more boredom and monotony, compared with those exposed to the sport-specific videos. This is in accordance with previous research showing that boredom and monotony may induce psychological and physiological states of under-arousal or be important components of mentally fatiguing tasks [13,14,35,36]. 

## 5. Conclusions

Our study showed that a 30 min video-based tactical task did not elicit mental fatigue and did not impair subsequent physical and technical performance in a soccer SSG. In accordance with previous suggestions [6,9,13,21], this represents a step toward ecological validity because video-based tactical activities are frequently adopted by the coaching staff to inform the entire team or individual players about the playing characteristics of opponents during the training week or before the match itself. Even though players do not always have to explicitly provide constant answers as in the current study, they are nevertheless required to maintain sustained attention and focus on tactical situations and solutions for a similar period of time, therefore exerting cognitive effort. However, coaches should be careful to expose players to these kinds of conditions before training sessions and matches, because even if the global perception of effort after the SSG was not affected, the mental effort required by the tactical task was significantly higher (Figure 4). Accordingly, previous research has shown that common daily activities such as the use of smartphones, social networks, and playing video games might elicit mental fatigue and impair subsequent performance [22].

In future studies, researchers are encouraged to investigate further ecological activities that may undermine performance and investigate the potential of the situational sports performance itself to be a mentally fatiguing task, given its highly perceptual, cognitive, and decision-making requirements and demands [6,8,9]. 

As in previous research investigating the effects of mental fatigue in SSG, the main limitation of the present study is the small sample size, which can be partly obviated by a crossover design [21]. Moreover, participants are usually recruited from the same team to avoid meaningful differences in short-term training history, season period and microcycles, and training status. Additional research is needed to allow the generalization of these findings on players with different technical levels [21]. Specifically, our participants did not perform this type of activity routinely during the training week or before matches. Therefore, recruiting participants from different cohorts (amateur or elite players) that might be more or less accustomed to cognitive tactical sessions is required to improve the generalizability of current findings.

## Figures and Tables

**Table 1:** Physical activity variables expressed as means ± SD. VT = video-based tactical condition; C = control condition; ES = effect size; a positive % value means a higher value in VT in respect to C.

Variable	VT	C	Mean Difference as %	ES (Cohen’s d)	*p* Value
**Total distance (m)**	1449.4 ± 81.6	1454.5 ± 116.0	−0.4%	0.05 very small	0.872
**Walking distance (m)**	657.6 ± 27.8	672.1 ± 54.1	−2.2%	0.31 small	0.343
**Low speed distance (m)**	657.4 ± 82.1	640.2 ± 126.1	+2.6%	0.15 very small	0.549
**High speed distance (m)**	124.2 ± 53.6	129.8 ± 47.9	−4.6%	0.11 very small	0.765
**Very high-speed distance (m)**	10.2 ± 9.5	12.4 ± 14.2	−21.4%	0.17 very small	0.745
**Total accelerations (n)**	31.0 ± 8.8	31.4 ± 6.5	−1.3%	0.05 very small	0.867
**Total decelerations (n)**	31.3 ± 8.4	30.6 ± 8.4	+2.2%	0.08 very small	0.819
**Playerload**	80.3 ± 8.2	80.0 ± 10.2	+0.4%	0.03 very small	0.814

**Table 2:** Technical variables expressed as means ± SD. VT = video-based tactical condition; C = control condition; ES = effect size. * = significant difference between conditions (*p* ≤ 0.05); a positive % value means a higher value in VT in respect to C.

Variable	VT	C	Mean Difference as %	ES (Cohen’s d)	*p* Value
**Total passes (n)**	23.0 ± 10.4	16.3 ± 7.3	+29.1%	0.72 medium	0.003 *
**Successful passes (n)**	19.7 ± 8.9	13.1 ± 6.8	+33.5%	0.82 large	0.003 *
**Negative passes (n)**	3.3 ± 2.5	3.2 ± 0.9	+3.0%	0.05 very small	0.868
**Passing accuracy (%)**	85.9 ± 7.4	77.5 ± 10.0	+9.9%	0.95 large	0.055
**Total tackles (n)**	7.3 ± 2.9	6.6 ± 1.8	+9.6%	0.28 small	0.588
**Successful tackles (n)**	5.1 ± 2.4	4.6 ± 1.9	+9.8%	0.2 small	0.668
**Negative tackles (n)**	2.2 ± 1.7	2.0 ± 1.4	+9.1%	0.13 very small	0.785
**Tackling success (%)**	69.5 ± 29.0	70.1 ± 21.6	−0.9%	0.02 very small	0.961
**Total shots (n)**	3.8 ± 3.1	3.5 ± 2.9	+7.9%	0.1 very small	0.671
**Successful shots (n)**	1.2 ± 1.7	0.7 ± 1.3	+41.7%	0.32 small	0.238
**Negative shots (n)**	2.6 ± 2.0	2.8 ± 2.1	−7.7%	0.1 very small	0.566
**Shooting accuracy (%)**	28.3 ± 34.1	13.2 ± 23.4	+53.5%	0.5 medium	0.249
**Control errors (n)**	1.6 ± 1.4	1.0 ± 1.2	+37.5%	0.46 small	0.340

## Data Availability

All data are presented and available within the manuscript. Data are also available from the Zenodo database. DOI: 10.5281/zenodo.6299602.
